# Prenatal Exposure to Bisphenol A: Is There an Association between Bisphenol A in Second Trimester Amniotic Fluid and Fetal Growth?

**DOI:** 10.3390/medicina59050882

**Published:** 2023-05-04

**Authors:** Nikolaos Loukas, Dionysios Vrachnis, Nikolaos Antonakopoulos, Vasilios Pergialiotis, Areti Mina, Ioannis Papoutsis, Christos Iavazzo, Alexandros Fotiou, Sofoklis Stavros, Georgios Valsamakis, Nikolaos Vlachadis, Georgios Maroudias, George Mastorakos, Zoi Iliodromiti, Petros Drakakis, Nikolaos Vrachnis

**Affiliations:** 1 Department of Obstetrics and Gynecology, Tzaneio General Hospital, 185 36 Piraeus, Greece; 2 Department of Clinical Therapeutics, Alexandra Hospital, Medical School, National and Kapodistrian University of Athens, 115 28 Athens, Greece; 3 Third Department of Obstetrics and Gynecology, Attikon Hospital, Medical School, National and Kapodistrian University of Athens, 124 62 Athens, Greece; 4 First Department of Obstetrics and Gynecology, Alexandra Hospital, Medical School, National and Kapodistrian University of Athens, 115 28 Athens, Greece; 5 Department of Forensic Medicine and Toxicology, Medical School, National and Kapodistrian University of Athens, 115 27 Athens, Greece; 6 Department of Gynecologic Oncology, Metaxa Memorial Cancer Hospital, 185 37 Piraeus, Greece; 7 Second Department of Obstetrics and Gynecology, Endocrine Unit, Aretaieion Hospital, Medical School, National and Kapodistrian University of Athens, 115 28 Athens, Greece; 8 Department of Obstetrics and Gynecology, Kalamata General Hospital, 241 00 Kalamata, Greece; 9 Department of Neonatology, Aretaieion Hospital, Medical School, National and Kapodistrian University of Athens, 115 28 Athens, Greece

**Keywords:** Bisphenol A, endocrine-disrupting chemical, inflammation, large for gestational age, small for gestational age, birth weight, excessive growth, amniotic fluid, fetal growth restriction, intrauterine growth restriction

## Abstract

*Background and Objectives:* Fetal growth abnormalities increase the risk of negative perinatal and long-term outcomes. Bisphenol A (BPA) is a ubiquitous endocrine-disrupting chemical to which humans may be exposed in a number of ways, such as from the environment, via various consumer products, and through the individual’s diet. Since the compound possesses estrogen-mimicking properties and exerts epigenetic and genotoxic effects, it has been associated with harmful effects impacting the entire spectrum of human life, including, vitally, the intrauterine period. We investigated the role of maternal exposure to BPA in abnormal fetal growth velocity, both impaired and excessive. *Materials and Methods:* Amniotic fluid samples were collected from 35 women who underwent amniocentesis early in the second trimester due to medical reasons. Pregnancies were followed until delivery, and birth weights were recorded. The amniotic fluid samples were subsequently divided into three groups based on fetal birth weight, as follows: AGA (appropriate for gestational age), SGA (small for gestational age), and LGA (large for gestational age). Amniotic fluid BPA levels were determined by gas chromatography coupled with mass spectrometry. *Results*: BPA was detected in 80% (28/35) of our amniotic fluid samples. Median concentration was 281.495 pg/mL and ranged from 108.82 pg/mL to 1605.36 pg/mL. No significant association was observed between the study groups regarding BPA concentration. A significant positive correlation between amniotic fluid BPA concentration and birth weight centile (r = 0.351, *p*-value = 0.039) was identified. BPA levels were also inversely associated with gestational age in pregnancies at term (between 37 and 41 weeks) (r = −0.365, *p*-value = 0.031). *Conclusions*: Our findings suggest that maternal exposure to BPA during the early second trimester of pregnancy can potentially contribute to increased birthweight percentiles and to decreased gestational age in pregnancies at term.

## 1. Introduction

Fetal growth disorders, namely impaired and excessive fetal growth, are considered to be consequential contributors to adverse perinatal and long-term outcomes. Small for gestational age (SGA) fetuses/neonates are defined as those with an estimated fetal weight (EFW) or birthweight (BW) below the 10th centile for gestational age, while large for gestational age (LGA) refers to fetuses/neonates with an EFW or BW above the 90th centile for gestational age [1]. SGA fetuses are further divided into constitutionally small fetuses due to maternal characteristics (height, weight, and ethnicity) and growth-restricted ones (FGR), the latter reflecting a pathological state of diminished growth and failure to reach the individualized predetermined growth potential. The cause of impaired growth can either be due to intrinsic (mainly genetic) diseases, infection, and maternal exposure to harmful factors or be placenta-mediated, mainly via impaired placentation and diminished nutrient transfer [2,3]. Similarly, excessive growth is attributable either to hereditary characteristics (maternal height and weight) or to pathological causes, such as maternal malnutrition, gestational diabetes, and genetic disorders, including Beckwith-Wiedemann and Sotos syndromes [1,4].

Fetal growth abnormalities have been linked to increased morbidity and mortality at all stages of life, according to the “fetal origin of adult disease” hypothesis [5]. Specifically, they have been correlated with stillbirth, perinatal asphyxia, neonatal mortality, hypothermia, hypoglycemia, prematurity, and higher risk for adverse outcomes in childhood and adulthood, among them neurodevelopmental defects, diabetes mellitus, cardiovascular diseases, and metabolic syndrome. LGA fetuses, in particular, are prone to birth injuries, such as brachial plexus injuries and shoulder dystocia, while their mothers are at increased risk for major postpartum hemorrhage and pelvic floor trauma. It is therefore crucial to identify factors predisposing to fetal growth disturbances as early in pregnancy as possible, thereby limiting their short- and long-term effects [6,7,8].

Endocrine-disrupting chemicals (EDCs) exist as both natural and man-made compounds that can interfere with metabolism, hormonal homeostasis, and the functioning of the endocrine system either by mimicking or by blocking the biological activity of endogenous hormones. They have been associated with adverse effects in both human and animal studies and are considered to be potentially harmful factors predisposing to abnormal fetal growth, since the majority of them are able to cross the protective placental barrier and enter the fetal circulation, as revealed by the detection of EDCs in fetal biological samples such as amniotic fluid and/or cord blood [9,10,11].

Bisphenol A (BPA), in particular, is one of the most ubiquitous EDCs. Utilized in the manufacture of polycarbonate plastics and epoxy resins, it is present in food packaging (food and beverage containers), plastic drinking bottles, toys, water pipes, medical devices, dental sealants, and thermal paper. However, the most common route of exposure is through food and drink consumption, as it can leach into the products when the protective coatings and bottles are overheated. Other routes of exposure include inhalation of air and dust and absorption through the skin [12].

BPA exerts endocrine-disrupting properties due to its estrogen-mimicking properties and its functional interference with thyroid and androgen hormones via several receptors and metabolic pathways, among them estrogen and estrogen-related transcription receptors, androgen, and thyroid hormone receptors. BPA can also interact with pregnane x, aryl hydrocarbon, and peroxisome proliferator-activated receptors [13,14]. The pregnane x receptor and aryl hydrocarbon receptors contribute to the detoxification of xenobiotics by regulating genes that encode enzymes participating in these processes. Peroxisome proliferator-activated receptors are a group of nuclear receptor proteins that participate in the regulation of triglyceride, glucose, and fatty acid metabolism, playing a crucial role in energy homeostasis and lipid metabolism. Moreover, BPA has been associated with genotoxicity, disruption of the meiotic process, and chromosomal aberrations, as well as epigenetic changes, such as histone modifications, DNA methylation, and genomic imprinting [15,16]. Given that pregnancy is considered to be a crucial period affecting the whole lifespan, prenatal exposure to this endocrine disruptor has been correlated with adverse perinatal and long-term outcomes, inter alia, miscarriage, preterm birth, disturbed fetal growth, neurodevelopmental deficits, reproductive pathologies, metabolic disorders, and obesity [17,18]. As mentioned above, numerous earlier studies have demonstrated the ability of BPA to cross the placenta and enter the fetal circulation, as established via its detection in amniotic fluid samples [19,20].

Prevention of abnormal fetal growth velocities today constitutes one of the most challenging issues in obstetrics. The first essential step is the early recognition of fetuses at risk. This can be achieved by identifying substances like BPA that possess the ability to affect normal fetal growth and predispose to impaired or excessive fetal growth, both conditions negatively affecting the perinatal and long-term fetal outcomes.

The aim of this study is to provide insights into the correlation between maternal BPA exposure and fetal growth.

## 2. Materials and Methods

Amniotic fluid samples were obtained from women undergoing amniocentesis in the early second trimester (15–22 weeks) due to medical indications, including increased risk for chromosomal abnormalities identified in the first- or second-trimester screenings, increased nuchal translucency, previous history of birth defects, and advanced maternal age, as well as on maternal request. Multiple pregnancies and pregnancies with major congenital abnormalities or abnormal karyotype were excluded from the study, while only full-term healthy singleton pregnancies with no pathologies that can affect fetal growth were included in the study. All pregnancies were followed until delivery.

The amniotic fluid samples were collected in polypropylene tubes to avoid any contamination and were stored at −80 °C until the day of BPA measurement. Identical collection materials were used for all samples. All cases were divided after birth into three study groups based on birth weight, namely, AGA, SGA, and LGA. Overall, our study included 35 amniotic fluid samples. Amniotic fluid BPA levels were determined by gas chromatography coupled with mass spectrometry.

Trial analysis for the determination of BPA in amniotic fluid samples was conducted at the Department of Forensic Medicine and Toxicology of the Medical School, National and Kapodistrian University of Athens, Athens, Greece. The chemicals, reagents, and laboratory equipment used are described in Table S1 of Supplementary Material.

Informed consent was given by all women participating. The Bioresearch and Ethics Committee of Attikon University Hospital, Athens, Greece approved the study (SC 10092019, approved on 10 September 2019).

### 2.1. Sample Preparation

For the determination of total BPA (free and conjugated with glucuronic acid) in the amniotic fluid samples, enzymatic hydrolysis was performed prior to extraction. Each sample (500 μL amniotic fluid) and procedural blank (500 μL water) was spiked with 50 μL of d_16_-BPA (internal standard) (10 ng/mL) and mixed for 10 s. A total of 300 μL of M sodium acetate buffer pH5 was added to each sample and mixed for 10 s in order to acidify the samples. Additionally, 25 μL (112.5 mU) of β-glucuronidase was added for the hydrolysis. The use of both 25 μL (112.5 mU) and of 50 μL (225 mU) of β-glucuronidase had been tested, and the former quantity was selected to ensure maximum deconjugation. The samples were mixed for 10 sec in vortex and then firmly tapped and incubated overnight at 37 °C. The next day, 2 mL of ethyl acetate was added: the mixtures were then mixed in vortex for 2 min, after which they were centrifuged at 3000 rpm for 5 min at room temperature (RT). After being transferred to clean glass incubation tubes, the supernatants containing the extracted BPA and internal standard were evaporated to dryness under a stream of N_2_ at RT. For derivatization, the samples were reconstituted with 50 μL of ethyl acetate and 50 μL of pentafluoropropionic anhydride (PFPA), firmly tapped, and incubated at 70 °C for 30 min. After the samples had been left to cool for 10 min at RT, they were again evaporated to dryness under a stream of N_2_. Lastly, they were reconstituted with 70 μL ethyl acetate and loaded in autosampler vials to be injected into the GC/MS system.

### 2.2. Gas Chromatography-Mass Spectrometry—Analysis of the System and Conditions

The mass spectrometer was operated in the electron impact (EI) ionization mode together with the selective ion monitoring (SIM) mode. Helium was employed as the carrier gas at a flow rate of 1 mL/min. A sample amount of 1 μL was injected in the splitless mode. GC program parameters were optimized in the following manner: with initial oven temperature at 100 °C, the temperature was maintained for 1 min and then increased at a rate of 30 °C/min to the maximum 300 °C, which was maintained for 5 min, the total runtime being 12.69 min. The injector, ion source, and interface temperatures were fixed at 250, 230, and 280 °C, respectively.

To estimate the mean calibration curve used in the quantification of the amniotic samples, each one of the three analytical runs included one procedural blank and six calibration spiked samples, prepared as described below, while the fourth run included one procedural blank and the amniotic samples of unknown concentration. Derivatized BPA and d_16_-BPA were retained for 6.05 and 6.03 min, respectively. The mass fragments used for the identification of BPA were *m*/*z*: 505, 506, and 520, and for d_16_-BPA, they were *m*/*z*: 516, 517, and 534.

### 2.3. Calibration Standard Solutions

Stock standard solutions of BPA and d_16_-BPA were prepared at a concentration of 1 mg/mL in methanol. Working standard solutions at final concentrations of 5 (solution A) and 100 ng/mL (solution B) for BPA and 10 ng/mL for d_16_-BPA were prepared by sequential dilutions of stock solutions with methanol. Using these working solutions, six calibration samples were prepared by spiking 500 μL of water with 50 μL of d_16_-BPA (10 ng/mL) and the corresponding volumes of working solutions A and B. The calibration samples were applied for the plotting of calibration curves (based on the peak area ratio of each analyte to the corresponding internal standard) together with the quantification of BPA in amniotic fluid samples. All stock and working standard solutions were stored at 4 °C.

### 2.4. Validation Data

BPA was not detected in any of the blank samples, and no co-eluting peaks were observed at the retention times of the derivatized BPA and d_16_-BPA. The rate of recovery of the method has been estimated to be 94–100%. Limit of quantification (LOQ), which is the lowest point of the calibration curve, was determined at 0.1 ng/mL, while limit of detection (LOD) was determined at 0.03 ng/mL. No samples with concentrations between LOQ and LOD were found. Linearity was determined by calculating the regression line using a least-squares method with a weighting factor of 1/x^2^ (where x is the concentration of analyte) and evaluated by the square of the correlation coefficient (R^2^). The method was linear in the concentration range 0.1–10 ng/mL, with a correlation coefficient of (R^2^) > 0.991.

### 2.5. Statistical Analysis

The results were analyzed using the SPSS statistical package (IBM Corp. Released 2012. IBM SPSS Statistics for Windows, Version 21.0. Armonk, NY, USA: IBM Corp). The comparison of BPA concentrations and other measured variables between the three groups was conducted by means of the Kruskal-Wallis test, a non-parametric method for comparing independent samples. The normality of distribution of sample values was evaluated with the Kolmogorov-Smirnov regression analysis test. Data that did not meet the criterion were analyzed using non-parametric methods. Median values and 25th–75th percentiles were calculated for each variable. Comparison of categorical variables between groups was conducted using the chi-square test. For the correlation between BPA concentration and other arithmetic parameters, we used Spearman’s correlation coefficient. All tests were two-sided, and we set the level of statistical significance at a p-value of less than 0.05. For amniotic fluid samples with BPA concentration below LOD, we imputed a standardized value of LOD divided by 2 (LOD/2).

## 3. Results

Our final study sample comprised 35 amniotic fluid specimens, including 16 in the AGA (control) group, 12 in the SGA group, and 7 in the LGA group. BPA could not be detected in seven cases with the method used in our study. All cases were matched for gestational age, fetal sex, maternal parity, age, weight, height, and smoking status. Table 1 illustrates maternal demographics and fetal characteristics for all three studied groups. The values for measured variables are presented as median values and 25th–75th percentiles and for categorical variables as percentages. The individual data for all 35 samples are provided in Table S1 in Supplementary Material.

No statistically significant difference was noted between the studied groups regarding maternal age, weight, height, parity, smoking status, fetal sex, and mode of delivery. Significant differences were observed in respect to gestational age, neonatal birth weight, and birth weight centile, the latter being expected since SGA and LGA fetuses are by definition respectively lighter and heavier than normal fetuses, while an earlier induced delivery may be elected for the extreme centiles.

Mean BPA concentration (in samples with BPA concentration above LOD) was 461.341 pg/mL, while median BPA concentration was 281.495, with a range of values between 108.82 pg/mL and 1605.36 pg/mL (minimum and maximum measured value). For all samples, including those with BPA concentration below LOD, mean concentration was 372.07 pg/mL, while median BPA concentration was 250 pg/mL.

**Figure 1:**
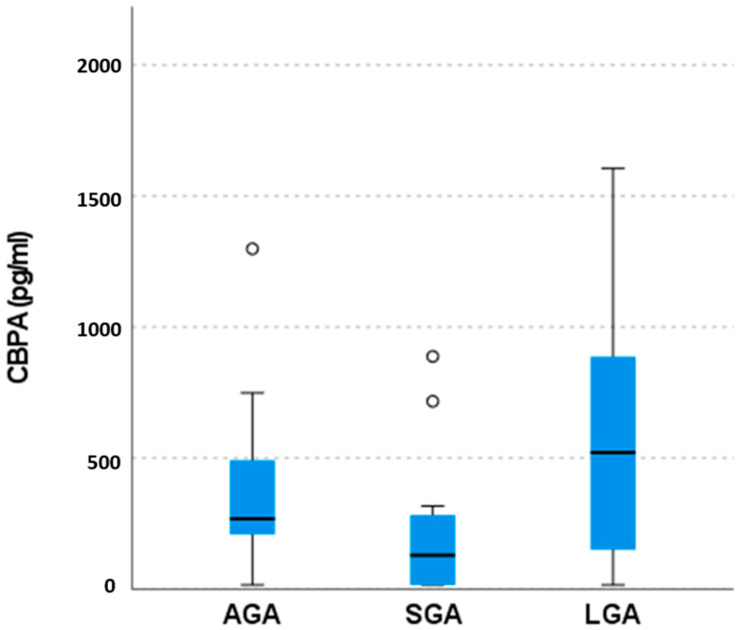
Amniotic fluid BPA concentration levels (CBPA) in the three studied groups (AGA, SGA, LGA) measured in pg/mL. The bold line in the boxes indicates median value, and box limits indicate 25th and 75th percentiles. Whisker plot limits show the extreme values measured in each group (minimum and maximum) excluding outliers (i.e., values more than the 1.5 interquartile range above the upper edge of the boxes). Outliers are designated with a circle. Regarding CBPA in the AGA group median value, 25th and 75th percentiles are 267.82 pg/mL, 206.81 pg/mL, and 503.27 pg/mL, respectively. For the SGA group CBPA median value, 25th and 75th percentiles are 128.62 pg/mL, 15 pg/mL and 299.42 pg/mL, respectively. For the LGA group median value, 25th and 75th percentiles are 520.59 pg/mL, 15 pg/mL and 1022.73 pg/mL, respectively.

Figure 1 summarizes BPA’s gas chromatography coupled with mass spectrometry analysis results, showing the median values and 25th–75th percentiles of BPA concentration in the amniotic fluid of the three studied groups (SGA, LGA, and AGA (control) groups). Higher BPA concentrations were observed in the LGA group compared to the AGA group, with median values of 520.59 pg/mL and 267.82 pg/mL, respectively. This difference, however, was not statistically significant (*p*-value = 1). Lower BPA concentrations were detected in the SGA group (median value of 128.62 pg/mL) compared to the AGA/control group and LGA group, although these differences failed to reach statistical significance (*p*-values are 0.296 and 0.188, respectively).

Table 2 displays the Spearman rank correlation coefficient between BPA concentration and all other numeric parameters (i.e., maternal age, maternal weight and height, gestational age, birth weight, and birth weight centile). BPA levels were positively correlated with birth weight (r = 0.260) and birth weight centile for gestational age (r = 0.351, *p*-value = 0.039), with the latter correlation being statistically significant. BPA concentration was also inversely correlated with gestational age in pregnancies at term (between 37 and 41 weeks) (r = −0.365, *p*-value = 0.031).

As sex-specific effects have been reported in previous studies, we conducted a further subgroup analysis for male and female fetuses (Table 3). Regarding female fetuses, we found no statistically significant results (Spearman’s correlation coefficient *p*-values > 0.05). On the contrary, BPA concentrations in the amniotic fluid of male fetuses were positively correlated with birth weight centile for gestational age (r = 0.451), a result that was marginally non-significant (*p*-value = 0.053), while they were also negatively correlated with gestational age (r = −0.627, *p*-value = 0.004), although this was not included in our primary outcomes to be investigated.

## 4. Discussion

Identifying factors contributing to disturbed fetal growth is of utmost importance and today comprises a promising field of research. External factors, such as maternal diet, lifestyle, and the environment, interfere with normal growth [18,21]. In the present study, we compared amniotic fluid BPA concentration in the second gestational trimester of normal fetuses with that of fetuses exhibiting extreme growth patterns (LGA, SGA). Our results showed no significant association between the studied groups. However, BPA concentration was significantly positively correlated with birth weight centiles and also inversely correlated with gestational age in pregnancies at term.

Regarding male fetuses, there is a marginally non-significant positive correlation between BPA concentration and birth weight centile along with a statistically significant negative correlation with gestational age, effects that are not observed among female fetuses. This result may be due to the variations in the differences between males and females as regards levels of endogenous sex hormones, which disparity is likely to alter responsiveness to EDCs. Another mechanism that can lead to different results based on gender is the differences in the androgen-related metabolism of BPA, as studies have shown that BPA is positively correlated with testosterone, and serum BPA is higher in normal adult men [22,23].

The median BPA concentration in our research (0.28 ng/mL) is similar to those reported in previous studies, with values ranging from 0.26 ng/mL to 1.03 ng/mL. The latter studies diverged with respect to their detection methods (Elisa, LC-ECAPCI-MS/MS, LC–MS, and GC-MS) and, consequently, as regards the limit of detection and quantification [19,20,24,25]. To our knowledge, our study is distinguished by having the lowest LOD (0.03 ng/mL) and LOQ (0.1 ng/mL) among all existing studies. BPA was detected in 80% of our samples (28/35). In the study of Edlow et al. [20], BPA was also detected in 80% of samples, whereas two other research groups had markedly lower detection rates (40% and 10%) [19,24].

Several epidemiological studies on the potential association between BPA and fetal growth have been conducted in the past few years, with many of them supporting our results. A prospective study in Korea pointed to a positive association between BPA measured in maternal urine in the third trimester of pregnancy and birth weight, with a z-score for birth weight and birth weight centile of male neonates and ponderal index of female neonates [26,27]. Other authors using ultrasound parameters as indicators of fetal growth showed that maternal urine BPA is positively related to head circumference in girls [28]. When exposure to BPA and its analogs (BPF, BPS) was measured, an increase in female infants’ ponderal index was observed, combined with a decrease in birth length [29].

In another study, both serum and cord blood BPA levels were also examined by two research groups who reported a positive correlation between their samples. These studies also demonstrated a positive association between birth weight and BPA levels in maternal serum and cord blood [30,31]. Other authors measured BPA at delivery and revealed a positive association with birth length, mainly in boys [32]. A meta-analysis conducted in 2019 that included 7 studies and 3004 participants also reported results similar to those of our study, showing that BPA has a significant positive association with birth weight in all neonates [33].

In contrast to the above results, other studies suggested either a negative association between BPA and birth weight or no relation at all. One study observed that BPA concentration in maternal blood in the highest quartile is correlated with increased risk for SGA and LBW male infants [34], while others using placental samples reported elevated concentrations in SGA and LBW neonates and a negative association with birth weight [35].

Regarding the inverse association between BPA exposure and gestational age, similar findings were reported in a systematic review and meta-analysis that included nine studies with urine BPA measurements [36].

The contradictory results may be attributed to the heterogeneity of the current studies given that different biological samples are used, collected from populations with different exposure levels and during different timeframes of pregnancy. In addition, there is a diversity of detection methods employed, with different LOQs, which, combined with the intrinsic characteristics of BPA (short half-life, high intra-individual variability), can lead to these conflicting outcomes between studies [37,38]. Moreover, since BPA may exert different effects when combined with other EDCs, the presence of other pollutants can modify the results, as mixtures of EDCs occasionally have either synergistic or antagonistic effects [39].

Over the last few years, discussion has been raised concerning the obesogenic effects of many substances, among them EDCs. BPA, in particular, displays obesogenic potency by accelerating adipogenesis, dysregulating adipokine secretion, and inducing inflammation. These changes are partially mediated by the action of BPA on estrogen receptors α (ERα) and β (ERβ), along with other receptors, such as the thyroid, peroxisome proliferator-activated receptor c (PPARc), glucocorticoid receptors, and the retinoid X receptor (RXR). Additionally, BPA exerts epigenetic effects during critical periods of life, such as the prenatal period and infancy. Elevated BPA levels in human and animal studies have been correlated with higher BMI or body weight at all stages of postnatal life. Furthermore, BPA can modulate glucose homeostasis and insulin resistance and has been associated with metabolic disorders, including, as mentioned, obesity, as well as diabetes mellitus. It is thus clear that the above chemical compounds are risk factors contributing to the delivery of LGA neonates [40,41,42].

Concerning its estrogen-mimicking properties, BPA is one of the most ubiquitous xenoestrogens to which the general population is exposed. Estrogenic activity is involved in adipogenesis and the process of cell proliferation, hence playing an essential role in fetal growth and development. Several studies have shown that maternal serum estrogen levels are positively correlated with fetal biometry and weight [43]. Meanwhile, BPA is a known modulator of two other metabolic regulators, leptin and adiponectin, leptin being a hormone mainly produced by adipose tissue. There is evidence that increased BPA exposure is correlated with higher maternal levels of leptin [44], and increased leptin levels are, in turn, associated with LGA infants, while elevated leptin and adiponectin levels in cord blood are associated with increased birth weight [45,46].

Finally, BPA has been linked to changes in normal placental structure. The placenta is the source of nutrients and oxygen supply for the fetus, while in parallel filtering out products, and is thus the principal regulator of fetal growth. An animal study showed that BPA oral administration to Sprague Dawley rats resulted in a higher ratio of fetal weight to placenta weight and in upregulation of glucose type 1 transporter (GLUT1), the main placental transporter that facilitates the transport of glucose to the fetus. The latter effect was also reported when human trophoblastic cells were treated with BPA, indicating a potential mechanism leading to excessive growth through increased glucose uptake [47]. In another animal study, rats orally treated with BPA exhibited reduced placental weight and reduced uterine artery diameter and vasodilatation properties. These findings suggest that maternal exposure to BPA increases the risk for endothelial dysfunction of uterine arteries, thereby playing a pivotal role in fetal growth and other pregnancy complications. Interestingly, fetal weight was found to be increased, this possibly being a compensatory action of the placenta given that BPA increases placental glucose transfer, as already mentioned [48]. Experimental studies have also shown signs of impaired angiogenesis and impaired placental development, changes that may be associated with preterm birth [49].

To our knowledge, our study is the first to compare BPA concentration levels in second trimester amniotic fluid of AGA, SGA, and LGA fetuses. Second trimester amniotic fluid, reflecting as it does the composition of fetal plasma and containing molecules and factors that facilitate fetal growth, is an appropriate fetal environment for assessment of fetal exposure to BPA [50,51,52]. On the other hand, BPA concentration in maternal plasma may not be representative of the true exposure of the fetus, as studies have shown that exposure depends on placental BPA permeability [24]. Nevertheless, an important strength of this study is that, as far as we know, it is distinguished by utilizing one of the lowest LODs and LOQs, including measurements of BPA levels in amniotic fluid; this determination is not incorporated in any other studies.

The main limitations of our study, as well as of other published studies, is the relatively small sample size and the fact that our sample was composed of amniotic fluid collected in the early second trimester, although the exposure of the fetus continued into the late second and third trimesters up until delivery. As a result, we were unable to ascertain the time interval at which the fetus is most susceptible to BPA exposure. Another major challenge was that BPA has a short half-life (approximately 6 h in maternal circulation), which can lead to estimation bias [37].

## 5. Conclusions

Given that BPA is a globally prevalent environmental contaminant, extra caution is needed to avoid high exposure during pregnancy, the prenatal period representing a time window of increased vulnerability for the fetus. Crucially, it appears evident that the compound can contribute to abnormal fetal growth velocity. The objective of this study is to provide insights into the correlation between maternal BPA exposure and fetal growth. Our findings strongly suggest that BPA concentration in second-trimester amniotic fluid is positively associated with birth weight centiles and inversely correlated with gestational age in pregnancies at term.

## Figures and Tables

**Table 1:** Maternal demographics and fetal characteristics of the studied groups (AGA, SGA, LGA).

	AGA	SGA	LGA	*p*-Value
Maternal age (years)	35.5 (29.5–39.75)	34 (31–36.75)	38 (36–39)	0.175
Maternal weight (kg)	60 (53–63)	57 (52.25–68.5)	61 (58–67)	0.563
Maternal height (cm)	163(158–170)	165 (161–167.75)	167 (167–172)	0.199
Nulliparity	9/16 (56.25%)	6/12 (50%)	1/7 (14.2%)	0.171
Non-smoking	12/16 (75)	7/9 (77.7%)	6/7 (85.7%)	0.874
Gestational age (years)	39 (38.7–39.6)	39.1 (38–39.5)	38 (37.4–38.1)	0.003
Neonatal birth weight (grams)	3325 (3135–3555)	2655 (2410–2795)	3800 (3700–3800)	<0.001
Neonatal sex (female)	8/15 (53.3%)	5/10 (50%)	0/7 (0%)	0.052
Mode of delivery–cesarean section	7/16 (43.75%)	6/12 (50%)	5/7 (71.4%)	0.559
Birth weight percentile	48.5 (34.25–66)	5.5 (1–7)	94 (92–96)	<0.001

**Table 2:** Spearman’s rank correlation coefficient between BPA concentration and all other arithmetic parameters. BPA concentration levels are positively correlated with birth weight centile for gestational age.

	BPA	Age	Weight	Height	Birth Weight	Gestational Age	Percentile
BPA	1	0.027	−0.060	0.151	0.260	−0.365 *	0.351 *
Maternal Age		1	0.002	0.024	0.201	0.059	0.157
Maternal Weight			1	0.458 **	0.031	−0.340 *	0.052
Maternal Height				1	0.074	−0.324	0.112
Birth weight					1	−0.167	0.974 **
Gestational age						1	−0.337 *
Percentile							1

* Correlation is significant at the 0.05 level; ** Correlation is significant at the 0.01 level.

**Table 3:** Spearman’s rank correlation coefficient between BPA concentration and all other arithmetic parameters according to gender of fetus. BPA concentration levels in the amniotic fluid of male fetuses are positively correlated with birth weight and birth weight centile for gestational age, while they are negatively correlated with gestational age. No correlation was found for female fetuses.

	Age	Weight	Height	Gestational Age	Birth Weight	Percentile
BPA-Males	−0.187	−0.127	−0.055	−0.627 **	0.320	0.451
BPA-Females	0.263	−0.512	0.152	0.176	0.272	0.383

** Correlation is significant at the 0.01 level.

## Data Availability

The data presented in this study are available on request from the corresponding author.

## Supplementary Materials

The following supporting information can be downloaded at: https://www.mdpi.com/article/10.3390/medicina59050882/s1, Table S1: Chemicals, reagents, and laboratory equipment.
