# Supplementary material for: Prenatal Exposure to Bisphenol A: Is There an Association between Bisphenol A in Second Trimester Amniotic Fluid and Fetal Growth?
Source: Medicina (Kaunas). 2023 May 4;59(5):882. doi: 10.3390/medicina59050882 (PMC10222006; doi:10.3390/medicina59050882)
Supplement: Supplementary file 1 [file medicina-59-00882-s001.zip › medicina-2170444-supplementary.pdf]

Table S1 *Chemicals, reagents, and laboratory equipment*

**Chemicals and reagents**	**Laboratory equipment**	**Equipment for gas chromatography coupled with mass spectrometry**
Bisphenol A (50 g, ≥99% purity, Sigma-Aldrich, Saint Louis, USA)	Reax Top vortex mixer (Heidolph, Schwabach, Germany)	GC/MSD model 6890N.05.05/5975 Inert XL MSD (Santa Clara, USA)
d16-bisphenol A (250 mg, ≥98% purity, Sigma-Aldrich, Saint Louis, USA)	Dry bath (Thermo Fisher Scientific, Waltham, USA)	Agilent 7683B series autosampler system (Santa Clara, USA)
β-glucuronidase/arylsulfatase (H. pomatia, 10 mL, Roche Diagnostics, Basel, Switzerland)	Gas evaporation system connected with nitrogen (N2) gas tank (Reacti-Vap Pierce Model 18780, Thermo Fisher Scientific, Waltham, USA)	Edwards vacuum pump (West Sussex, UK)
Acetone (HPLC grade, Merck, Darmstadt, Germany)	691 digital pHmeter with glass electrode (Metrohm, Herisau, Switzerland)	DB-5MS chromatography column, 30 m x 0.25 mm i.d x 0.25 µm film thickness (Agilent, Santa Clara, USA)
Methanol (HPLC grade, Merck, Darmstadt, Germany)	Precision balance (Radwag, Radom, Poland)	Chemstation software (Hewlett Packard, California, USA)
Ethyl acetate (HPLC grade, Merck, Darmstadt, Germany)	Heraeus Labofuge 400R centrifuge (Thermo Fisher Scientific, Waltham, USA)	
Water (HPLC grade, Sigma-Aldrich, Saint Louis, USA)	Automatic pipette 0.2-1.0 mL (Pipetman Classic, Gilson, Middleton, USA), with appropriate tips	
Pentafluoropropionic anhydride (PFPA, 25g 99%, Sigma-Aldrich, Saint Louis, USA)	Automatic pipette 0.05-0.2 mL (Pipetman Classic, Gilson, Middleton, USA), with appropriate tips	
1 M Sodium acetate buffer pH 5 (6.8 g of sodium acetate and 3 mL glacial acetic acid in 0.5 L H2O, adjustment to pH 5 with 1N HCl)	10 mL glass centrifugation tubes	
	15 mL glass incubation tubes	
	Glass volumetric pipettes (5.00, 10.00 mL)	
